# Associations of Objective Functional and Subjective Health Literacy with Reproductive Health Knowledge and Preconception Preparedness Among Pregnant Women in Hungary

**DOI:** 10.3390/medsci14060692

**Published:** 2026-10-21

**Authors:** Ilona Karácsony, Tímea Völgyiné Bujdosó, Réka Vajda, Diána Elmer, Annamária Pakai, Zsuzsanna Kívés

**Affiliations:** 1 Department of Prevention and Perinatal Medicine, Institute of Basics of Health Sciences, Midwifery and Health Visiting, Faculty of Health Sciences, University of Pécs, H-7621 Pecs, Hungary; 2 County Health Visitor Service Department, Győr-Moson-Sopron County Petz Aladár University Teaching Hospital, H-9422 Harka, Hungary; vedono.harka@petzkorhaz.hu; 3 Institute of Health Insurance, Faculty of Health Sciences, University of Pécs, H-7621 Pecs, Hungary; reka.vajda@etk.pte.hu (R.V.); diana.elmer@etk.pte.hu (D.E.); zsuzsa.kives@etk.pte.hu (Z.K.); 4 Institute of Emergency Care, Pedagogy of Health and Nursing Sciences, Faculty of Health Sciences, University of Pécs, H-7621 Pecs, Hungary; annamaria.pakai@etk.pte.hu

**Keywords:** health literacy, reproductive health, preconception preparedness, pregnancy, pregnant women, prenatal care

## Abstract

**Background/Objectives:** Adequate reproductive health knowledge and preconception preparedness may support informed health-related decisions during pregnancy and before conception. Health literacy may influence the ability to access, understand, and use health information; however, its association with reproductive health knowledge and preconception preparedness among pregnant women remains insufficiently explored. This study assessed objective functional and subjective health literacy among pregnant women in Hungary and examined their associations with a composite measure of reproductive health knowledge and preconception preparedness after adjustment for sociodemographic and obstetric characteristics. **Methods:** This cross-sectional study included 346 pregnant women in Hungary. Objective functional and subjective health literacy were assessed using the Newest Vital Sign (NVS) and Brief Health Literacy Screening Tool (BRIEF), respectively. The primary outcome was a researcher-developed, non-validated composite measure comprising four theoretically distinct domains: nutrition and lifestyle knowledge during pregnancy, smoking-related knowledge, preconception preparedness, and knowledge of risks associated with advanced maternal and paternal age. Domain scores were standardised to a 0–100 scale and equally weighted. Associations were examined using chi-square tests, Kruskal–Wallis tests, analysis of variance, and hierarchical multiple linear regression. In the final model, BRIEF and NVS scores were entered simultaneously as continuous variables, with adjustment for age, educational attainment, residence, gestational age, parity, pregnancy planning, and financial situation. **Results:** Composite scores differed significantly across objective functional health literacy categories (F(2, 343) = 9.618; *p* < 0.001; η^2^ = 0.053), with higher scores observed among participants with adequate objective functional health literacy. Subjective health literacy was also associated with the composite score (F(2, 343) = 4.93; *p* = 0.008; η^2^ = 0.028). In the fully adjusted hierarchical regression model, objective functional health literacy was positively associated with the composite outcome ((B = 1.732; SE = 0.553; β = 0.271; *p* = 0.001), whereas subjective health literacy was not statistically significantly associated with the outcome (B = 0.244; SE = 0.203; β = 0.088; *p* = 0.227). The simultaneous addition of both health literacy measures increased explained variance by 7.7 percentage points (ΔR^2^ = 0.077; ΔF = 7.121; *p* = 0.003), with the final model explaining 15.1% of the variance. **Conclusions:** Objective functional health literacy was positively associated with the composite measure of reproductive health knowledge and preconception preparedness among pregnant women in Hungary, whereas subjective health literacy was not significantly associated with the outcome after simultaneous adjustment. The findings indicate that objective and subjective health literacy capture partly different aspects relevant to reproductive health-related knowledge and preparedness. Given the cross-sectional design and the non-validated, researcher-developed outcome measure, causal conclusions cannot be drawn. Further research using validated multidimensional instruments and longitudinal or intervention designs is warranted.

## 1. Introduction

The health awareness and health-related behaviours of women of reproductive age, particularly pregnant women, are of central importance for maternal, fetal, and neonatal health outcomes [1]. Pregnancy is a critical life stage during which health-related decisions and behaviours become especially important. Accordingly, the literature conceptualises pregnancy as a classic “teachable moment.” Pregnancy may strengthen intrinsic motivation to change health behaviours, thereby creating a favourable opportunity to adopt risk-reducing behaviours and establish a healthy lifestyle that can be sustained in the longer term [2,3]. However, behaviour change is a complex process in which, beyond motivation, individuals’ psychological and practical capabilities, as well as the social and environmental opportunities available to them, are also decisive. According to the COM-B model, motivation to change alone is therefore insufficient for the adoption of appropriate health behaviours; individuals also need the capability to access and understand health-related information [2].

One key component of these capabilities is health literacy, which encompasses the ability to access, understand, critically appraise, and apply health information in everyday health-related decision-making [4,5]. This is particularly important during pregnancy, when women face increased information and decision-making demands, while effective collaboration with healthcare providers and appropriate interpretation of received information may be directly related to maternal and fetal health [6,7]. Adequate health literacy enables pregnant women not only to obtain health information but also to interpret it, assess the reliability of different sources, communicate effectively with healthcare professionals, and apply information to decisions concerning their own health [7].

Health literacy is increasingly conceptualised as a multidimensional construct extending beyond basic reading and numeracy skills. Sørensen et al. proposed an integrated model that describes health literacy in terms of the knowledge, motivation, and competencies required to access, understand, appraise, and apply health-related information across healthcare, disease prevention, and health promotion. This framework emphasises that health literacy involves not only understanding information but also critically evaluating it and applying it in everyday health-related decision-making. These dimensions are particularly relevant during pregnancy, when women are required to interpret health information, assess potential risks, and make informed decisions concerning nutrition, preventive care, and maternal and fetal health [8].

An important methodological feature of health literacy research is that health literacy can be measured using both subjective and objective functional approaches. Subjective health literacy measures are based on individuals’ self-assessments of their own abilities and perceived difficulties in processing health information, whereas objective functional instruments typically assess functional reading comprehension and numeracy skills. These approaches do not necessarily yield identical classifications, suggesting that individuals’ perceptions of their health literacy may differ from their objectively measurable functional skills [9,10]. This issue is particularly relevant in Hungary, where available evidence indicates that a substantial proportion of the adult population has limited health literacy skills, while notable discrepancies have also been observed between self-reported measures and indicators assessing practical health literacy [9]. These findings highlight the need to examine health literacy in pregnant populations from a multidimensional perspective.

Recent studies have further highlighted the relevance of health literacy during pregnancy and the importance of considering both its prevalence and its relationship with health-related behaviours. In a cross-sectional study, Sajjadian et al. examined the relationship between health literacy and components of preconception care among pregnant women. Their findings indicated statistically significant associations between health literacy and several preventive practices, including preconception care, folic acid supplementation, blood testing, and cervical cancer screening [11]. These findings suggest that health literacy may be relevant to the uptake of preventive services and health-promoting practices before and during early pregnancy.

Evidence from Hungary also indicates that health literacy difficulties may be common among pregnant women. Csölle et al. reported that 50.8% of participants had limited health literacy according to the Newest Vital Sign (NVS), while 46.7% were classified as having limited health literacy using the Brief Health Literacy Screening Tool (BRIEF). These findings illustrate that the estimated prevalence of health literacy difficulties may vary according to the assessment instrument used. They also underline the importance of examining health literacy using complementary measurement approaches in pregnant populations [12].

The prevalence and distribution of health literacy difficulties have also been examined in other settings. In a large cross-sectional study conducted in the United Arab Emirates, Elbarazi et al. found that higher educational attainment, employment, and adequate social support were associated with higher odds of adequate health literacy among pregnant women [13]. In Qatar, Naja et al. reported that inadequate health literacy was common among pregnant women. Their analyses also identified associations between inadequate health literacy and sociodemographic characteristics, including educational attainment and household income [14]. Taken together, these studies highlight the importance of considering health literacy in the context of pregnancy while taking into account differences in measurement instruments, population characteristics, and social context.

Limited health literacy may be particularly problematic during pregnancy, as it has been associated with healthcare utilisation, health behaviours, and health-related decision-making. Research suggests that pregnant women with lower health literacy may experience greater difficulties navigating the healthcare system and obtaining, understanding, and applying appropriate health information [15,16]. Limited health literacy has also been associated with later initiation of antenatal care and the inappropriate use of pregnancy-related dietary supplements [17,18]. Similarly, pregnant women with lower health literacy may be less likely to use folic acid appropriately and to access antenatal care [19].

The relationship between health literacy and specific reproductive health knowledge and preconception preparedness score therefore warrants particular attention. Available studies suggest that lower health literacy may be associated with knowledge gaps related to family planning, contraception, pregnancy planning, and the risks associated with advanced maternal age [19,20,21]. Knowledge deficits have also been identified in relation to smoking and nutrition. Women with lower health literacy may be more likely to underestimate the risks of smoking, while their knowledge regarding appropriate nutrition before and during pregnancy and the required micronutrients may also be insufficient [19,20,22]. This is especially relevant because factual knowledge alone does not guarantee its appropriate application in practice; the ability to critically appraise and personalise information is also necessary for optimal health behaviour [7].

However, the association between health literacy and reproductive health knowledge cannot be interpreted independently of social background factors. Several studies have indicated that educational attainment, economic circumstances, and place of residence may play important roles in shaping health literacy levels [10,21,23]. Limited health literacy has been observed more frequently among individuals with lower educational attainment, less favourable financial circumstances, occupations requiring lower qualifications, and in certain rural areas [24,25]. Studies conducted among pregnant populations have reported similar associations, with educational attainment, economic circumstances, place of residence, age, and selected health-related behaviours being related to maternal health literacy [26,27,28]. Nevertheless, not all studies have identified significant associations between health literacy and sociodemographic variables, highlighting the context-dependent nature of these relationships and the importance of measurement approaches [29].

Overall, previous findings indicate that health literacy is an important resource during pregnancy, supporting the processing of health information, the adoption of appropriate health behaviours, and the use of healthcare services. However, much of the existing research has primarily linked health literacy to health behaviours, healthcare utilisation, or risk perception. Less is known about the extent to which objectively and subjectively assessed health literacy is associated with specific reproductive health knowledge and preconception preparedness. Evidence also remains limited on whether these associations persist after adjustment for sociodemographic and obstetric characteristics.

Addressing this research gap, the present study examines whether objective functional and subjective health literacy among pregnant women are associated with reproductive health knowledge and preconception preparedness after simultaneous adjustment. The study further extends beyond a single knowledge domain by integrating several areas relevant to pregnancy, including nutrition, smoking-related risks, informed family planning, and risks associated with advanced maternal and paternal age. The simultaneous use of two distinct health literacy measures also allows examination of whether pregnant women’s self-perceived health literacy and objectively assessed functional skills are similarly associated with reproductive health knowledge and preconception preparedness.

The practical relevance of this study lies in examining whether health literacy is associated with reproductive health knowledge and preconception preparedness after adjustment for selected sociodemographic and obstetric characteristics. Such findings may help identify areas for further research on health literacy-sensitive antenatal education. This could contribute to the development of targeted, needs-based educational strategies that are informed not only by pregnant women’s sociodemographic characteristics but also by their actual information-processing and health-related decision-making needs.

The following hypotheses were formulated:

**H1.**  *Pregnant women’s sociodemographic characteristics—age, educational attainment, and place of residence—are significantly associated with both objective functional and subjective health literacy levels.*

**H2.**  *Different levels of objective functional and subjective health literacy are associated with different levels of reproductive health knowledge and preconception preparedness. Pregnant women with adequate health literacy achieve higher overall reproductive health knowledge and preconception preparedness scores.*

**H3.**  *After simultaneous adjustment for sociodemographic and obstetric characteristics, objective functional and subjective health literacy are each positively associated with the reproductive health knowledge and preconception preparedness score.*

## 2. Materials and Methods

### 2.1. Study Design and Participants

This quantitative cross-sectional study included pregnant women who had completed the 13th week of gestation and were in their second or third trimester. Eligible participants were recruited through online communities and social-media platforms using non-probability convenience sampling and participated voluntarily. Women with prior education or professional training in healthcare were excluded because professional background could have influenced the outcomes. Additional exclusion criteria were pregnancy achieved through assisted reproductive technology and questionnaires with unprocessable missing data.

Because recruitment was conducted online and participation was voluntary, the sample was not intended to be population-representative. The sampling approach may have resulted in selection bias, particularly with respect to educational attainment, socioeconomic circumstances, access to online health information, and engagement with pregnancy-related online communities. Accordingly, the findings should be interpreted as associations observed within the study sample rather than as population estimates for pregnant women in Hungary.

A total of 367 questionnaires were received. Of these, 21 were excluded because they were not sufficiently completed to allow evaluation of the main study variables. The final analytical sample therefore comprised 346 pregnant women (*N* = 346). No a priori sample-size or statistical power calculation was performed. The final analytical sample was determined by the number of eligible and sufficiently complete responses obtained through the voluntary online recruitment strategy.

### 2.2. Data Collection and Measures

Data were collected using an anonymous, self-administered online questionnaire. Participants were recruited through targeted online communities and social media platforms addressing reproductive health, family planning, pregnancy, and motherhood among women with young children. In addition to sociodemographic and obstetric background variables, the questionnaire included validated health literacy measures and researcher-developed, non-validated measures of reproductive health knowledge and preconception preparedness designed for the purposes of the present study.

Background variables included age, educational attainment, self-rated financial situation, place of residence, gestational age, number of children, and pregnancy planning status.

Health literacy was assessed using two standard instruments validated in Hungarian. Objective functional health literacy was measured using the validated Hungarian version of the Newest Vital Sign (NVS) [30]. This instrument assesses reading comprehension and numeracy skills in a health-related context. Participants answered six questions based on a food nutrition label. Each correct response was awarded one point, yielding a maximum score of 6. Based on total scores, participants were classified as having inadequate (0–1 points), limited (2–3 points), or adequate (4–6 points) health literacy. For selected categorical association analyses, the inadequate and limited categories were combined into a below-adequate category because of the small number of participants in the inadequate category. The adequate category was retained separately. Associations between objective functional health literacy and categorical sociodemographic variables were subsequently examined using 2 × 2 contingency tables. Pearson’s chi-square test was used when the expected cell frequencies met the assumptions of the test. Cramér’s V was reported as a measure of association.

Subjective health literacy was assessed using the Hungarian validated version of the four-item Brief Health Literacy Screening Tool (BRIEF) [31,32]. Participants rated each item on a five-point Likert scale. Based on the total score calculated by summing the items, participants were classified as having inadequate (4–12 points), marginal (13–16 points), or adequate (17–20 points) health literacy. Internal consistency was acceptable in the present sample (Cronbach’s α = 0.78).

### 2.3. Reproductive Health Knowledge and Preconception Preparedness Assessment

Reproductive health knowledge and preconception preparedness were assessed using a researcher-developed, non-validated instrument designed specifically for the present study and informed by protective and risk factors identified in the literature. No formal expert-panel review or pilot psychometric validation was undertaken before data collection. The primary outcome was operationalised as a study-specific composite measure of reproductive health knowledge and preconception preparedness.

The assessment comprised four theoretically distinct domains: (1) nutrition and lifestyle during pregnancy, (2) smoking-related risks during pregnancy, (3) conscious preparation for pregnancy, and (4) risks associated with advanced maternal and paternal age.

The four domains were not assumed to represent a unidimensional psychometric construct. Rather, they were selected to cover complementary areas of reproductive health relevant to pregnancy and preconception care. Importantly, the conscious preparation domain included both knowledge-related content and self-reported preconception practices, such as lifestyle modification, healthcare consultation, screening, and folic acid or prenatal vitamin use. Therefore, this domain should be interpreted as a broader indicator of knowledge-related preparation and practice rather than as a pure measure of factual knowledge.

Because the instrument has not undergone comprehensive construct or criterion validation, the domain scores and the overall composite score should be interpreted as study-specific indicators rather than as validated measures of reproductive health knowledge and preconception preparedness.

Knowledge of healthy nutrition and lifestyle during pregnancy was assessed researcher-developed objective knowledge test. Items were scored according to a predefined scoring key. The scoring rule is study-specific and is not presented as a clinical contraindication. Depending on the structure of the item, individual questions contributed one or more points to the total score. Single-response items contributed one point for a correct answer, whereas items requiring the identification of multiple correct responses allowed one point for each correctly identified response. The question assessing the recommended increase in daily energy intake during the second and third trimesters contributed a maximum of two points, with one point awarded for each correctly identified trimester-specific value [33,34]. The food-avoidance/moderation item contributed a maximum of four points [35], and the open-ended omega-3 source item a maximum of three points [36]. The question concerning the effects of alcohol consumption during pregnancy contributed a maximum of two points [37,38]. The questionnaire included an open-ended item assessing participants’ awareness of sweeteners whose consumption during pregnancy they considered to require particular consideration. Participants were presented with a list including aspartame, sorbitol, saccharin, mannitol, acesulfame-K, fructose and lactose, as well as a “do not know” response option. For the purpose of the study-specific scoring, aspartame, saccharin and acesulfame-K were included in the predefined scoring key. The items concerning foods to be avoided or moderated during pregnancy distinguished between food-safety-related recommendations and dietary recommendations concerning moderation. Raw or inadequately cooked meat, foods containing raw egg, and products made from unpasteurised milk were considered food-safety-related items. The item concerning meat prepared in a large amount of added fat was intended to assess recognition of the recommendation to limit high-fat, energy-dense foods rather than to identify a specific food-safety hazard [35]. Incorrect or non-scored responses received zero points. The researcher-developed nutrition knowledge items were reviewed against relevant scientific literature and evidence-based professional recommendations concerning nutrition and health-related behaviours during pregnancy. The scoring approach was revised accordingly. The item concerning sweeteners was excluded from the scored nutrition knowledge assessment because its wording did not allow for sufficiently unambiguous scoring. The nutrition knowledge score and the statistical analyses affected by this modification were recalculated using the revised scoring procedure. The theoretical maximum subscore was 16 points. Internal consistency of this knowledge test was acceptable (Cronbach’s α = 0.788).

Knowledge of fetal and neonatal health risks related to active smoking, second-hand smoke exposure, and third-hand smoke exposure during pregnancy was assessed using a 13-item, researcher-developed questionnaire [39,40,41]. Participants rated statements on a five-point Likert scale (1 = strongly disagree; 5 = strongly agree). Reverse scoring was applied to negatively worded items and items reflecting misconceptions. The global smoking knowledge score, derived by summing responses, had a maximum of 65 points. The scale showed high internal consistency (Cronbach’s α = 0.87).

Knowledge of conscious preparation for pregnancy was examined using a six-item, researcher-developed question set. The extent of preconception lifestyle modification was evaluated on a five-point Likert scale. Additional points were awarded for specific areas of lifestyle modification; consultation with a healthcare professional before conception; participation in preconception screening; screening related to genetic conditions, pregnancy suitability and sexually transmitted infections; and deliberate folic acid or prenatal vitamin supplementation before conception [42,43]. The maximum composite score was 14 points and showed acceptable internal consistency in the present sample (Cronbach’s α = 0.705).

Knowledge of risks associated with childbearing at advanced maternal and paternal age was assessed using five researcher-developed questions. The items assessed knowledge of maternal age-related risks and threshold ages, maternal complications, and risks and complications associated with paternal age [44,45,46]. Based on the sum of correct response components from closed-ended single- and multiple-choice questions, a subscore ranging from 0 to 17 points was calculated, with higher scores indicating greater knowledge.

### 2.4. Composite Reproductive Health Knowledge and Preconception Preparedness Score

Overall reproductive health knowledge and preconception preparedness was operationalised across four theoretically distinct knowledge domains: (1) nutrition and lifestyle knowledge during pregnancy, (2) knowledge of smoking-related risks during pregnancy, (3) knowledge of conscious preparation for pregnancy, and (4) knowledge of risks associated with advanced maternal and paternal age. The preconception preparedness domain included self-reported behavioural and healthcare-use indicators, including lifestyle modification, preconception consultation, screening, and folic acid or prenatal vitamin use. It was therefore not considered a pure measure of reproductive health knowledge. Similarly, the smoking-related domain assessed knowledge-related beliefs and perceptions using Likert-type agreement ratings rather than objective factual knowledge items.

Because the domains comprised different numbers of items and maximum possible scores, directly summing the raw subscores would have resulted in unequal contributions of the individual domains to the overall score. To avoid this, each subscore was standardised according to its theoretical maximum score. This standardisation was performed to place the four domains on a common metric and to prevent domains with larger raw score ranges from contributing disproportionately to the summary measure. Domain-specific scores were transformed to a 0–100 scale using the following formula:

$$\mathrm{Standardised domain score}=\left(\frac{\mathrm{Obtained score}}{\mathrm{Maximum possible score}}\right)\times 100$$

The overall composite score was then calculated as the arithmetic mean of these four standardised scores, giving each domain an equal weight of 25%. This equal-weighting approach represents a predefined conceptual decision to provide a transparent summary indicator, rather than a psychometrically derived weighting scheme. Consequently, the resulting 0–100 score should be interpreted as an exploratory, study-specific multidimensional indicator rather than a validated unidimensional scale. To avoid relying exclusively on this composite measure, domain-specific analyses were also retained. The theoretical range of the composite score was 0–100 points, with higher values indicating greater reproductive health knowledge and preconception preparedness.

The following formula was applied:

$$\mathrm{RHK}=\frac{T+S+P+A}{4}$$

where

RHK = overall reproductive health knowledge and preconception preparedness score.

*T* = standardised nutrition and lifestyle knowledge score.

*S* = standardised smoking-related knowledge score.

*P* = standardised preconception/conscious preparation knowledge score.

*A* = standardised knowledge score for risks associated with advanced maternal and paternal age.

### 2.5. Statistical Analysis

Statistical analyses were conducted using IBM SPSS Statistics version 30.0. Microsoft Excel 2016 was used for data preparation and verification.

The primary analysis examined the association between the overall reproductive health knowledge-preconception preparedness score and health literacy using hierarchical multiple linear regression. Analyses of individual knowledge domains were considered exploratory and were interpreted as domain-specific, hypothesis-generating analyses.

For descriptive analyses, means (Ms), standard deviations (SDs), medians, interquartile ranges (IQRs), minimum and maximum values were calculated as appropriate, whereas absolute and relative frequencies were calculated for categorical variables. The distribution of continuous variables was assessed using histograms, Q–Q plots, summary statistics, and the Kolmogorov–Smirnov test as a supplementary assessment. For comparisons involving three or more independent groups, one-way analysis of variance (ANOVA) was used when the assumptions of approximate normality and homogeneity of variance were sufficiently met; otherwise, the Kruskal–Wallis test was applied. Following a statistically significant ANOVA, Tukey’s HSD test was used for post hoc pairwise comparisons. Following a statistically significant Kruskal–Wallis test, Dunn’s test with Bonferroni correction was used for pairwise comparisons. Effect sizes were reported as η^2^ for ANOVA and ε^2^ for Kruskal–Wallis analyses where applicable. Chi-square tests were used to assess associations between categorical variables, with Cramér’s V reported as an effect-size measure.

Internal consistency was assessed for domains for which calculation of Cronbach’s alpha was considered appropriate. The resulting coefficients were interpreted as indicators of internal consistency only and not as evidence of content, construct or criterion validity.

H3 was examined using hierarchical multiple linear regression, with the overall reproductive health knowledge and preconception preparedness score entered as the dependent variable, to assess whether subjective and objective functional health literacy were statistically associated with the primary outcome after adjustment for relevant sociodemographic and obstetric characteristics. In Step 1, age, educational attainment, place of residence, gestational age, parity, pregnancy planning status, and financial situation were entered as control variables. In Step 2, subjective health literacy (BRIEF score) and objective functional health literacy (NVS score) were entered simultaneously as continuous variables. This specification allowed the associations of the two health literacy measures with reproductive health knowledge to be estimated while accounting simultaneously for the selected sociodemographic and obstetric characteristics and for the overlap between the two health literacy measures.

Educational attainment was coded dichotomously (0 = secondary education; 1 = higher education). Place of residence was represented by two dummy variables, using the capital city/county seat as the reference category. Pregnancy planning status and financial situation were similarly dummy coded, with planned prengancy and good financial situation serving as the respective reference categories.

Model fit was evaluated using R^2^ and adjusted R^2^, while the additional explained variance associated with Step 2 was assessed using ΔR^2^ and the corresponding ΔF statistic. Standardised regression coefficients (β) and 95% confidence intervals were used to describe the magnitude and precision of associations. Because the outcome score was bounded between 0 and 100, the regression findings were interpreted as adjusted statistical associations rather than causal or predictive effects.

Sensitivity analyses were conducted to examine the robustness of the regression findings to alternative specifications of the composite outcome. Two alternative outcome scores were constructed using the same 0–100 standardisation procedure as for the primary composite. In the first sensitivity analysis, the preconception preparedness domain was excluded, and the outcome was calculated from the remaining three domains. In the second sensitivity analysis, the nutrition knowledge domain was excluded, and the outcome was calculated from the remaining three domains. For both sensitivity analyses, hierarchical linear regression models were fitted using the same covariates and the same simultaneous entry of BRIEF and NVS as in the primary model.

The assumptions of the hierarchical linear regression models were assessed using several diagnostic procedures. Linearity and homoscedasticity were examined using scatterplots of standardized residuals against standardized predicted values. The distribution of residuals was additionally assessed using histograms and normal probability plots. Multicollinearity was evaluated using variance inflation factors (VIFs), and influential observations were examined using Cook’s distance. Standardized residuals were inspected for potentially extreme observations. The same diagnostic procedures were applied to the sensitivity models.

We also addressed the concern regarding information loss due to categorisation. In the revised primary regression analysis, both NVS and BRIEF scores are entered as continuous variables rather than categorical health literacy groups. This approach avoids reliance on the very small inadequate-NVS subgroup and preserves the information contained in the original scores. The association between subjective and objective functional health literacy was examined using Spearman’s rank correlation coefficient because the NVS is a bounded 0–6 score with a limited number of possible values.

Statistical significance was set at *p* < 0.05.

### 2.6. Ethical Considerations

The study was approved by the Regional Research Ethics Committee of the University of Pécs Clinical Centre (approval no. 146-PTERKEB 2026/1). Data collection was voluntary and anonymous. Before starting the questionnaire, participants received information about the purpose and procedures of the study, as well as data-processing arrangements. By commencing the questionnaire, participants consented to the use of their data for research purposes in aggregated form.

Data processing complied with Regulation (EU) 2016/679 of the European Parliament and of the Council [47]. The study was conducted in accordance with the ethical principles of the Declaration of Helsinki.

## 3. Results

### 3.1. Sociodemographic Characteristics of the Sample

The participants had a mean maternal age of 30.49 years and a mean gestational age of 28.38 weeks, representing a predominantly highly educated and financially secure sample where 71.97% hold a university degree and 90.75% experience no financial difficulties. Geographically, the sample is balanced across different settlement types, with nearly 60% of the women expecting their first child (Table 1).

### 3.2. Levels of Objective Functional and Subjective Health Literacy and Their Sociodemographic Correlates

The mean score on the BRIEF scale, which assessed subjective health literacy, was 15.75 (SD = 3.79; range: 4–20). For subjective health literacy, 57 participants (16.47%) were classified as having inadequate, 126 (36.42%) as having marginal, and 163 (47.11%) as having adequate subjective health literacy.

Based on the Newest Vital Sign (NVS), which was used as an objective measure of objective functional health literacy, the mean participant score was 4.96 (SD = 1.37; range: 1–6). Six participants (1.73%) were classified as having inadequate objective functional health literacy, 51 (14.74%) as having limited health literacy, and 289 (83.53%) as having adequate objective functional health literacy.

Associations between objective functional and subjective health literacy and the examined sociodemographic factors were analysed separately. A statistically significant but weak association was found between type of residence and subjective health literacy (χ^2^ (4) = 13.24; *p* = 0.010; Cramér’s V = 0.138). The proportion of participants with adequate subjective health literacy was highest among those living in the capital city or a county seat (57.25%), compared with those living in towns (40.17%) or villages/rural municipalities (42.27%). Type of residence was not significantly associated with objective functional health literacy when the inadequate and limited NVS categories were combined into a below-adequate category (χ^2^(1) = 0.108, *p* = 0.742, Cramér’s V = 0.018).

Educational attainment was also significantly, although weakly, associated with subjective health literacy (χ^2^ (2) = 8.56; *p* = 0.014; Cramér’s V = 0.157). The proportion of participants with adequate subjective health literacy was 51.00% among those with higher education and 39.56% among those with secondary education. Inadequate subjective health literacy was reported by 21.98% of participants with secondary education and 13.25% of those with higher education. Similarly, educational attainment was not significantly associated with objective functional health literacy after combining the inadequate and limited NVS categories into a below-adequate category (χ^2^(1) = 0.007, *p* = 0.934, Cramér’s V = 0.004). All expected cell counts were at least 5.

The distribution of age differed significantly from normality (*p* = 0.005); therefore, health literacy groups were compared using the Kruskal–Wallis test. Age did not differ significantly across either subjective health literacy categories (H(2) = 2.08; *p* = 0.354) or objective functional health literacy categories (H(2) = 1.96; *p* = 0.375).

Overall, H1 received only partial support: educational attainment and place of residence were significantly associated with subjective health literacy, whereas neither was significantly associated with objective functional health literacy, and age was not significantly associated with either health literacy measure.

### 3.3. Reproductive Health Knowledge and Preconception Preparedness

#### 3.3.1. Knowledge of Nutrition During Pregnancy

The nutrition-related knowledge test had a maximum attainable score of 16 points. In the assessment of knowledge regarding healthy nutrition during pregnancy, the highest proportion of correct responses concerned the importance of folic acid supplementation: 96.82% of participants correctly identified folic acid as a vitamin that reduces the risk of neural tube defects. Basic knowledge regarding energy intake during pregnancy was also high: 98.27% correctly rejected the statement that pregnant women need to “eat for two.” Overall, 28.03% of the sample were familiar with the Hungarian Okostányér^®^ (“Smart Plate”) dietary guideline, a national food-based guide that uses a plate model to communicate recommendations on food groups, portion proportions, hydration, and healthy lifestyle practices [48]. Among those aware of the recommendation, 35.05% reported usually following it, 38.14% followed it occasionally, 19.59% rarely, and 7.22% never. The main recommended daily proportions of food groups in the Okostányér^®^ guideline were correctly identified by 47.11% of the full sample. Regarding foods and food-preparation practices that should be avoided or limited during pregnancy, participants were asked to identify relevant items from a list. The proportion correctly identifying the respective responses varied across items. In particular, 23.12% correctly identified deep-frying meat in a large amount of fat as a food-preparation practice that should be avoided or limited during pregnancy. Based on qualitative categorisation of responses to an open-ended question regarding dietary sources of omega-3 fatty acids that support fetal nervous system development, 80.06% of participants mentioned fish and fish oils; 57.23% mentioned nuts, seeds, and vegetable oils; and 12.43% mentioned vegetables and fruits as relevant sources. Knowledge related to alcohol consumption was high: 97.98% identified zero alcoholic drinks per day as the only harmless amount during pregnancy. Of the two correct statements concerning the long-term fetal effects of alcohol use during pregnancy and the risk of fetal alcohol syndrome, at least one was selected by 89.60% of participants. Among those providing at least one correct answer, 90.32% selected one correct statement and 9.68% selected both correct statements.

The mean total nutrition knowledge score was 10.38 out of a maximum of 16 points (SD = 2.24; range: 5–15). Score distribution differed significantly from normality (*p* < 0.001). No statistically significant differences in nutrition knowledge scores were observed across subjective health literacy categories (H(2) = 5.31; *p* = 0.091).

In contrast, nutrition knowledge scores differed significantly across objective functional health literacy categories (H(2) = 33.29; *p* < 0.001), with a moderate effect size (ε^2^ = 0.086). Median scores were 7 points (IQR = 5–8) in the inadequate category, 9 points (IQR = 6–12) in the limited category, and 11 points (IQR = 10–15) in the adequate category. Post hoc pairwise comparisons using Dunn’s test with Bonferroni correction showed that nutrition knowledge scores were significantly lower in the inadequate category than in the adequate category (*p* < 0.001) and in the limited category than in the adequate category (*p* < 0.001). No statistically significant difference was observed between the inadequate and limited categories after multiplicity adjustment (*p* = 0.099).

#### 3.3.2. Smoking-Related Knowledge

Basic knowledge of fetal risks associated with smoking during pregnancy was high. Overall, 94.20% of participants strongly agreed that smoking during pregnancy is harmful to the fetus. Regarding the transplacental passage of nicotine and carbon monoxide, 86.13% strongly agreed and an additional 9.87% somewhat agreed. The association between smoking and spontaneous abortion, preterm birth, and stillbirth was strongly endorsed by 77.76% of participants, while a further 13.29% somewhat agreed. The increased risk of placental abruption was strongly recognised by 66.47%, and 16.76% somewhat agreed. The increased risk of cleft lip and palate was strongly recognised by 43.93%, with 15.09% somewhat agreeing.

Overall, 74.57% strongly agreed that smoking is associated with low birth weight, whereas 50.00% strongly recognised the increased risk of sudden infant death syndrome (SIDS). For the reverse-worded item on passive smoking, 54.62% strongly disagreed with the statement that environmental tobacco smoke does not harm the fetus. The association of passive smoking with preterm birth and ectopic pregnancy was strongly endorsed by 45.68%. The association between active and passive smoking and later asthma and allergic diseases in children was strongly recognised by 52.89%.

Lower proportions of correct responses were observed for alternative tobacco products and third-hand smoke exposure. Direct fetal risks of e-cigarette use were recognised by 74.57% of participants, whereas the risks of exposure to second-hand e-cigarette aerosol were recognised by 54.62%. The statement relating to third-hand smoke was strongly rejected by 41.91% of participants.

The mean total smoking-related knowledge score was 55.76 (SD = 7.18; range: 30–65). Score distribution was non-normal (*p* < 0.001). No significant differences were found across subjective health literacy categories (H(2) = 2.81; *p* = 0.245). However, smoking-related knowledge differed significantly across objective functional health literacy categories (H(2) = 6.69; *p* = 0.030), although the effect size was small (ε^2^ = 0.014). Median scores were 52 (IQR = 51–56) in the inadequate category, 56 (IQR = 50–61) in the limited category, and 57 (IQR = 52–62) in the adequate category. Post hoc pairwise comparisons using Dunn’s test with Bonferroni correction showed that none of the pairwise differences remained statistically significant after adjustment for multiple comparisons (inadequate vs. limited: *p* = 0.992; inadequate vs. adequate: *p* = 0.296; limited vs. adequate: *p* = 0.112).

#### 3.3.3. Knowledge of Conscious Preparation for Pregnancy and Preconception Preparedness

A planned pregnancy was reported by 92.77% of participants. The degree of lifestyle change before conception showed a heterogeneous distribution. Overall, 31.50% of participants reported no lifestyle changes before conception, while 15.61% reported minor, 25.43% moderate, 19.65% substantial, and 7.80% very substantial lifestyle changes.

The mean number of lifestyle domains modified was 1.28 (SD = 1.03; range: 0–4). The most reported change was adopting a healthier diet (77.64%), followed by initiating regular physical activity (38.40%). Complete avoidance of alcohol was reported by 46.84%, complete smoking cessation by 19.41%, and a reduction in smoking intensity by 4.22%.

Preconception consultation with a healthcare professional was reported by 31.79% of participants. Among these participants, consultation with an obstetrician-gynaecologist was most common (92.73%), followed by consultation with a general practitioner (15.45%) and a dietitian (11.82%). Only 2.73% of participants received preconception counselling from a health visitor. Of the preconception screening examinations, 73.12% had attended a general gynaecological examination and 18.50% a dental check-up. Overall, 23.71% had not undergone any screening examination before pregnancy. Screening for specific genetic conditions, pre-pregnancy fitness, or sexually transmitted infections was reported by 9.54%. Preconception use of a prenatal vitamin or folic acid supplementation was reported by 68.50%.

The mean score on the 14-point scale assessing conscious preparation for pregnancy was 5.83 (SD = 2.72; range: 1–13). Score distribution differed significantly from normality (*p* < 0.001). According to the Kruskal–Wallis test, conscious preconception preparation scores did not differ significantly across subjective health literacy categories (H(2) = 2.12; *p* = 0.340) or objective functional health literacy categories (H(2) = 1.52; *p* = 0.470).

#### 3.3.4. Knowledge of Risks Associated with Advanced Maternal and Paternal Age

Regarding knowledge of advanced maternal age, 97.69% of participants correctly identified that advanced maternal age may increase the risk of adverse pregnancy outcomes, and 87.28% correctly identified the age threshold at which risk increases. Among specific maternal and fetal risks, increased risks of chromosomal abnormalities (89.02%) and spontaneous miscarriage (76.30%) were recognised most frequently. Recognition rates were moderate for gestational hypertension (58.38%) and gestational diabetes mellitus (54.91%), whereas lower proportions identified increased risks of preterm birth (39.31%), caesarean delivery (32.08%), low birth weight (15.03%), and severe postpartum haemorrhage (12.43%).

Knowledge of risks associated with advanced paternal age was lower. Overall, 28.90% of participants believed that paternal age does not present a risk. Of the risks associated with paternal age, reduced likelihood of conception was recognised by 75.72% and deteriorating sperm quality by 60.98% of participants. Increased risk of congenital anomalies was identified by 34.39%, potential long-term health consequences for offspring by 16.76%, and paternal-age-related risks of spontaneous miscarriage and preterm birth by only 6.36%.

Participants achieved a mean score of 8.51 out of a maximum of 17 points on the knowledge test (SD = 2.91; range: 3–17). The Kruskal–Wallis test showed a significant difference across subjective health literacy categories (H(2) = 16.17; *p* < 0.001), with a small-to-moderate effect size (ε^2^ = 0.041). Median scores were 8 in both the inadequate and marginal subjective health literacy categories (IQR = 6–9 and 6–10, respectively), compared with 9 in the adequate category (IQR = 7–10). Post hoc pairwise comparisons using Dunn’s test with Bonferroni correction demonstrated that knowledge scores were significantly higher in the adequate category than in either the inadequate category (*p* = 0.001) or the marginal category (*p* = 0.007). No statistically significant difference was found between the inadequate and marginal categories (*p* = 0.783).

In contrast, knowledge scores did not differ significantly across objective functional health literacy categories (H(2) = 5.48; *p* = 0.062).

#### 3.3.5. Associations Between Reproductive Health Knowledge-Preconception Preparedness and Health Literacy

The mean composite reproductive health knowledge and preconception preparedness score, calculated as an equally weighted composite of the standardised subscores across the four knowledge domains, was 60.61 (SD = 9.72) points (range: 36.25–91.06). According to the Kolmogorov–Smirnov test, the score distribution did not differ significantly from normality.

A significant between-group difference was identified according to subjective health literacy, F(2, 343) = 4.93; *p* = 0.008; η^2^ = 0.028. The adequate subjective health literacy (M = 61.74, SD = 9.74) had significantly higher reproductive health knowledge and preconception preparedness scores than those in the inadequate group (M = 57.09, SD = 8.73) (MD = 4.65; 95% CI: 1.07–8.21; *p* = 0.006). No significant differences were found between the inadequate and marginal groups or between the marginal and adequate groups.

Overall reproductive health knowledge and preconception preparedness also differed significantly across objective functional health literacy categories (F(2, 343) = 9.618; *p* < 0.001; η^2^ = 0.053). Participants with adequate objective functional health literacy had higher mean knowledge scores than those with limited objective functional health literacy (61.66 vs. 55.75; mean difference = 5.91; 95% CI: 2.47–9.27; *p* < 0.001). No statistically significant differences were observed between the inadequate and limited groups or between the inadequate and adequate groups.

Table 2 summarises the results of analyses examining associations between reproductive health knowledge-preconception preparedness and health literacy.

### 3.4. Associations of Health Literacy with Reproductive Health Knowledge and Preconception Preparedness

A hierarchical multiple linear regression analysis was conducted to examine the associations of subjective and objective functional health literacy with overall reproductive health knowledge and preconception preparedness while controlling for sociodemographic and obstetric characteristics (Table 3).

In Step 1, age, educational attainment, place of residence, gestational age, parity, pregnancy planning status, and financial situation were entered as control variables.

The control-only model was not statistically significant (F(8, 337) = 1.664; *p* = 0.106) and explained 7.8% of the variance in reproductive health knowledge and preconception preparedness (R^2^ = 0.078; adjusted R^2^ = 0.036).

In Step 2, subjective health literacy (BRIEF score) and objective functional health literacy (NVS score) were entered simultaneously as continuous variables. The addition of the two health literacy measures significantly increased the explained variance (ΔR^2^ = 0.077; ΔF =7.121; *p* = 0.003). The final model explained 15.1% of the variance in reproductive health knowledge and preconception preparedness (R^2^ = 0.151; adjusted R^2^ = 0.103).

In the fully adjusted model, objective functional health literacy was positively associated with reproductive health knowledge and preconception preparedness (B = 1.732; SE = 0.553; β = 0.271; *p* = 0.001). In contrast, subjective health literacy was not statistically significantly associated with reproductive health knowledge and preconception preparedness after simultaneous adjustment for objective functional health literacy and the control variables (B = 0.244; SE = 0.203; β = 0.088; *p* = 0.227).

Among the control variables, residence in a village or rural municipality was negatively associated with reproductive health knowledge and preconception preparedness compared with residence in the capital city or a county seat (B = −4.046; SE = 1.804; β = −0.176; *p* = 0.029). Parity showed a borderline association with reproductive health knowledge and preconception preparedness (B = −1.530; SE = 0.863; β = −0.142; *p* = 0.056). Age, educational attainment, gestational age, pregnancy planning status, and financial situation were not statistically significantly associated with reproductive health knowledge and preconception preparedness in the final model.

Diagnostic assessment of the final regression model did not indicate substantial violations of the assumptions of multiple linear regression. The standardized residuals were approximately normally distributed based on visual inspection of the histogram and normal P–P plot. Standardized residuals ranged from −2.202 to 2.799, and studentized deleted residuals ranged from −2.287 to 2.891. No substantial multicollinearity was identified. VIF values ranged from 1.070 to 1.387 in the final model and from 1.107 to 1.386 in the control-only model. The maximum Cook’s distance was 0.051, and the Durbin–Watson statistic was 2.096, suggesting no indication of substantial autocorrelation or highly influential observations.

We also examined the association between the two health literacy measures. Spearman’s rank correlation showed a weak positive correlation between subjective and objective functional health literacy (ρ = 0.115; *p* = 0.033). This finding indicates that the two measures were statistically related but showed only limited overlap.

Taken together, the regression findings indicate that objective functional health literacy was associated with reproductive health knowledge-preconception preparedness after adjustment for the expanded set of sociodemographic and obstetric variables, whereas the association with subjective health literacy was attenuated after simultaneous inclusion of the objective measure. The findings therefore provide partial support for H3.

### 3.5. Sensitivity Analyses

Two sensitivity analyses were conducted to examine the robustness of the association between objective functional health literacy and the study-specific composite outcome. First, the preconception preparedness domain was excluded from the composite. Objective functional health literacy remained positively associated with the resulting outcome (B = 2.274, β = 0.377, 95% CI [1.533, 3.014], *p* = 0.001). Second, the nutrition and lifestyle knowledge domain was excluded to address potential content overlap between the NVS and the nutrition-related component of the composite. Objective functional health literacy remained positively associated with the alternative outcome (B = 1.238, β = 0.161, 95% CI [0.505, 2.027], *p* = 0.001). Thus, the positive association persisted across both alternative outcome specifications, although its magnitude varied depending on the domains included in the composite (Supplementary Table S1).

Inspection of residual-versus-predicted plots did not indicate substantial departures from linearity or homoscedasticity in the sensitivity models. The maximum absolute standardized residuals were 2.681 and 2.647, respectively, and the maximum Cook’s distance values were 0.030 and 0.031, indicating no evidence of highly influential observations. VIF values ranged from 1.087 to 1.386 in the first sensitivity model and from 1.090 to 1.372 in the second, providing no indication of problematic multicollinearity.

## 4. Discussion

The present study examined the associations between subjective and objective functional health literacy and reproductive health knowledge and preconception preparedness among pregnant women in Hungary. The findings indicated that the association between health literacy and reproductive health knowledge and preconception preparedness varied according to the health literacy measure and knowledge domain examined. Although both subjective and objective health literacy were associated with the overall knowledge score in unadjusted group comparisons, only objective functional health literacy remained statistically significantly associated with reproductive health knowledge and preconception preparedness after simultaneous adjustment for both health literacy measures and the expanded set of sociodemographic and obstetric variables. The magnitude of the final model also indicates that health literacy represents only one of several factors associated with reproductive health knowledge.

Objective functional and subjective health literacy capture distinct constructs. Whereas the NVS assesses the application of functional skills through specific tasks, the BRIEF reflects self-perceived health literacy. Based on BRIEF scores, 47.11% of respondents in the present study were classified as having adequate subjective health literacy, whereas 36.42% had marginal and 16.47% had inadequate subjective health literacy. In the Hungarian general population, Bíró et al. identified 34.3% with inadequate, 35.9% with marginal, and only 29.8% with adequate subjective health literacy [10]. Thus, the proportion of participants with adequate subjective health literacy was higher in the present sample; nevertheless, more than half of participants did not reach the adequate category.

The NVS, an objective measure of functional health literacy, indicated a more favourable profile: 83.53% of participants had adequate functional health literacy, 14.74% had limited health literacy, and only 1.73% were classified as having inadequate health literacy. These findings suggest a generally high level of functional health literacy in the study sample. The finding is notable in comparison with previously reported data from the Hungarian adult population, in which 68% had adequate, 20% limited, and 12% inadequate objective functional health literacy [9]. In the present sample, therefore, the proportion classified as adequate was substantially higher, whereas the combined proportion with inadequate or limited health literacy was lower.

In our study, there was a significant difference in the proportion of participants classified as having adequate health literacy based on the two assessment tools (BRIEF: 47.11% vs. NVS: 83.53%). This discrepancy is consistent with literature indicating that the BRIEF, as a subjective screening tool, captures individuals’ self-perceived navigation of health information, whereas the NVS objectively evaluates performance-based numeracy and reading skills, meaning they reflect distinct constructs of health literacy rather than contradictory findings.

This more favourable health literacy profile may partly reflect the characteristics of the study population, including frequent contact with the healthcare system during pregnancy and the high proportion of participants with higher education. The findings also indicate that health literacy may not function as a uniform construct during pregnancy. High functional skill levels do not necessarily imply that women perceive themselves as equally capable of managing health information.

When interpreting health literacy levels in the study population, it is important to consider the substantial heterogeneity reported across international studies. The prevalence of inadequate or limited health literacy among pregnant women has varied considerably across countries and populations, with sociocultural and economic contexts, as well as differences in assessment instruments, contributing to this variation. Therefore, rather than directly comparing prevalence estimates, it is more appropriate to compare overall patterns [19,32]. For example, Guler et al. in Turkey classified 66.1% of pregnant women as having limited health literacy, whereas only 33.9% had adequate health literacy [49]. Similarly, in a 2024 study by Cifci et al., 29.3% of women in the third trimester had inadequate, 46.2% problematic, 23.3% adequate, and only 0.9% excellent health literacy [26]. In Iran, Charoghchian Khorasani et al. identified adequate health literacy in 42.8% of pregnant women and inadequate health literacy in 15.5% [50]. In the study by Villadsen, approximately 33% of pregnant participants had sufficient health literacy, while only 10–20% achieved an excellent level [51].

The social composition of the sample may also be relevant to the interpretation of the results. A relatively high proportion of participants had higher education, which may provide an advantage in seeking and interpreting pregnancy-related information and navigating the healthcare system. Several previous studies support this assumption. In a Portuguese study, Ferreira et al. observed a higher prevalence of limited health literacy among younger women, women with lower educational attainment, and women experiencing financial difficulties; lower education, occupations requiring lower qualifications, and financial difficulties were identified as significant predictors of health literacy [25]. Hungarian and international studies have likewise identified educational attainment as a frequently reported social determinant of health literacy [9,10,25].

However, Vicente-Díaz et al. found no statistically significant association between health literacy and place of residence, age, marital status, or educational attainment [29]. This suggests that the role of sociodemographic factors may vary across populations and according to the health literacy measure used. In the present study, adequate subjective health literacy was significantly more common among participants living in the capital city or a county seat and among those with higher education, whereas age was not a differentiating factor.

Analysis of individual domains further nuanced these associations. Objective functional health literacy was particularly associated with knowledge of nutrition and smoking-related risks during pregnancy. Objective functional health literacy showed a statistically significant overall association with nutrition knowledge, with post hoc analyses indicating higher scores among participants in the adequate category than among those in the inadequate and limited categories. Although smoking-related knowledge differed significantly across objective functional health literacy categories in the overall Kruskal–Wallis analysis, none of the pairwise differences remained statistically significant after Bonferroni adjustment. Subjective health literacy was not significantly associated with nutrition knowledge, and smoking-related risks during pregnancy.

The results concerning smoking-related knowledge are particularly noteworthy. Most participants were aware of the basic fetal risks of active smoking: 94.20% strongly agreed that smoking during pregnancy is harmful to the fetus, and 86.13% correctly recognised that nicotine and carbon monoxide cross the placenta. However, recognition of more specific risks was less clear. Only 54.62% strongly rejected the statement that passive smoking is not harmful, and the same proportion considered exposure to second-hand e-cigarette aerosol harmful. Awareness of the risks of third-hand smoke—harmful substances remaining on clothing and furniture—was even lower, with only 41.91% of the sample recognising these risks. These findings suggest that widely communicated and familiar health messages may be better recognised than less well-known, specific risk information. Similarly, Oechsle et al., in a German cross-sectional study, found that pregnant women generally had good knowledge of smoking during pregnancy but often underestimated specific health effects [22].

Neither health literacy measure was significantly associated with knowledge of conscious preparation for pregnancy. This finding suggests that adequate general health literacy does not necessarily ensure specific knowledge across all areas of reproductive health. Reproductive health knowledge and preconception preparedness may therefore partly represent domain-specific knowledge, potentially influenced by encounters with healthcare services, targeted education, and prior experience.

Knowledge of risks related to advanced parental age was more strongly associated with subjective health literacy. Participants with adequate subjective health literacy achieved higher knowledge scores. This may suggest that, for more complex or less routine information relevant to reproductive decision-making, perceived competence in seeking and evaluating information may be more important than functional information-processing skills alone.

In the fully adjusted hierarchical regression model, objective functional health literacy was positively associated with reproductive health knowledge and preconception preparedness (B = 1.732; SE = 0.553; β = 0.271; *p* = 0.001), whereas subjective health literacy was not statistically significantly associated with the outcome (B = 0.244; SE = 0.203; β = 0.088; *p* = 0.227). Thus, the association observed for subjective health literacy in the unadjusted analyses was attenuated after simultaneous adjustment for objective functional health literacy and the additional control variables.

The two health literacy measures were only weakly positively correlated (Spearman’s ρ = 0.115; *p* = 0.033). This suggests that subjective and objective functional health literacy were related but showed limited overlap in the present sample. Accordingly, the two measures should not be interpreted as interchangeable indicators of the same construct.

The addition of the two health literacy measures to the control variables significantly increased the explained variance by 7.7 percentage points (ΔR^2^ = 0.077; ΔF = 7.121; *p* = 0.003). The final model explained 15.1% of the variance in reproductive health knowledge and preconception preparedness (adjusted R^2^ = 0.103). In the fully adjusted model, objective functional health literacy remained positively associated with the outcome, whereas subjective health literacy was not statistically significantly associated with the outcome after simultaneous adjustment. These findings should not be interpreted as demonstrating that objective functional health literacy has a greater causal or explanatory contribution than subjective health literacy; rather, they indicate that the two measures showed different patterns of association when considered simultaneously. Although this represents a statistically significant improvement in model fit, the relatively modest proportion of explained variance indicates that health literacy represents only one of several factors potentially associated with reproductive health knowledge.

The expanded regression model also showed an adjusted association between place of residence and reproductive health knowledge. Participants living in villages or rural municipalities had lower knowledge scores than those living in the capital city or a county seat (B = −4.046; SE = 1.804; β = −0.176; *p* = 0.029). This association should be interpreted cautiously and does not imply a causal effect of place of residence.

The present findings add to the growing evidence that health literacy is relevant to health-related knowledge and preventive behaviours during pregnancy. Previous research has reported associations between health literacy and the uptake of preconception care, folic acid supplementation, and selected preventive services [11]. Studies conducted among pregnant women have also highlighted the frequency of health literacy difficulties and the relevance of sociodemographic factors, including education, employment, and social support [12,13,14]. In the present study, objective functional health literacy was independently associated with the study-specific composite outcome, whereas subjective health literacy was not statistically significant in the adjusted model. This difference may indicate that self-perceived difficulties in processing health information and performance on an objective functional assessment capture partly distinct aspects of health literacy. However, given the cross-sectional design and the study-specific nature of the composite outcome, these findings should be interpreted as associations rather than evidence of a causal relationship.

The distinction between the two health literacy measures is particularly relevant when interpreting these findings. The BRIEF assesses subjective perceptions of health literacy, whereas the NVS provides an objective assessment of functional health literacy. Their weak correlation and their different patterns of association with reproductive health knowledge and preconception preparedness suggest that these measures capture different aspects of health-related competence.

The findings suggest that antenatal educational strategies may benefit from addressing specific reproductive health knowledge and preconception preparedness alongside broader aspects of health literacy. Gyulai et al. similarly argued that future strategies for reproductive health education should place greater emphasis on developing specific health literacy [52]. Identifying and addressing specific knowledge gaps is also important. Brief, structured assessments of knowledge and health literacy during early antenatal care may help identify domains requiring additional education. Interventions may therefore benefit from plain language, visual communication tools, and information delivery tailored to individual needs. Ferreira et al. and Karácsony et al. have likewise highlighted the potential value of brief health literacy assessments integrated into early antenatal care, plain-language communication, and visual tools, particularly for supporting groups with greater needs [25,53].

Antenatal care may therefore benefit from extending beyond the delivery of information to support its comprehension, interpretation, and application. This is particularly relevant to reproductive health decisions, in which both overestimation and underestimation of risks may lead to unfavourable decisions. Multidisciplinary antenatal care programmes and preventive interventions designed to improve health knowledge may consequently play an important role in supporting appropriate risk appraisal and informed risk management [54]. Pregnancy may provide an opportunity to influence behaviours such as nutrition, physical activity, and engagement with healthcare services, all of which can benefit maternal and child health [55,56].

The practical relevance of the present findings lies primarily in the potential value of integrating health literacy measurement and the assessment of reproductive health knowledge and preconception preparedness into the preventive approach of antenatal care. As pregnancy involves regular contact with healthcare professionals, it may offer a favourable opportunity for health promotion and educational interventions. Rather than relying solely on generic information provision, education may be tailored to individual health literacy levels and specific knowledge gaps.

Favourable general health literacy does not necessarily imply adequate knowledge across every reproductive health topic. Consequently, interventions need not be limited to socially disadvantaged groups; instead, all women receiving antenatal care should have access to understandable and reliable information, while more intensive support may be offered to those for whom limited health literacy or gaps in specific knowledge indicate a greater need.

For future programmes, a personalised, multi-stage education model could be considered. Such a model could begin with a brief assessment of health literacy and relevant knowledge domains, followed by the identification of individual gaps and the provision of tailored education. Regular interaction with the healthcare system and continuous education during pregnancy may increase opportunities for targeted interventions and support long-term improvements in health knowledge and health-related behaviours. Consideration of different stages of pregnancy may also be important. In a longitudinal study [7], Rockliffe et al. found that certain periods, particularly weeks 12–16 and 36–40, may provide especially favourable opportunities to influence health behaviours, including dietary behaviour [3].

The findings provided partial support for H3. Objective functional health literacy remained positively associated with reproductive health knowledge and preconception preparedness in the fully adjusted model, whereas subjective health literacy did not show a statistically significant association after adjustment for relevant sociodemographic and obstetric characteristics.

The relatively modest R^2^ of the final model also warrants consideration. Although the inclusion of health literacy significantly improved the model, 84.9% of the variance in reproductive health knowledge remained unexplained. Other factors not assessed in the present study, including previous healthcare experiences, exposure to reproductive health information, information-seeking behaviour, previous pregnancy-related experience, and educational or social influences, may also contribute to differences in reproductive health knowledge.

The sensitivity analyses further support the robustness of the association between objective functional health literacy and the composite outcome. The positive association between NVS scores and the outcome remained statistically significant when either the preconception preparedness domain or the nutrition knowledge domain was excluded from the composite score. Notably, the association persisted after removal of the nutrition domain, suggesting that the observed relationship was not solely attributable to content overlap between the NVS and the nutrition knowledge component. However, the magnitude of the association varied across the alternative outcome specifications. These findings provide additional support for interpreting objective functional health literacy as an aspect associated with reproductive health knowledge and preconception preparedness, while also highlighting the multidimensional nature of the study-specific outcome measure.

This study has several limitations that should be considered when interpreting the findings. Given the cross-sectional design, the observed associations cannot establish temporal ordering or causality. It cannot be determined whether higher objective functional health literacy contributes to greater reproductive health knowledge, whether greater knowledge is associated with better performance on objective functional health literacy tasks, or whether both are influenced by other unmeasured factors. Therefore, the findings should be interpreted as adjusted statistical associations rather than evidence that health literacy determines, leads to, or causally explains reproductive health knowledge. No a priori sample-size or statistical power calculation was performed, and the final sample size was determined by the number of eligible and sufficiently complete responses obtained through the voluntary online recruitment strategy.

The sampling strategy also limits the generalisability of the findings. Participants were recruited voluntarily through online communities and social media platforms related to pregnancy and reproductive health. This recruitment approach may have resulted in self-selection and digital-access bias and may have been more likely to reach women who were already engaged with health-related information. The sample was characterised by a high proportion of participants with higher education and favourable financial circumstances, as well as a high proportion with adequate objective functional health literacy. Therefore, the findings should not be considered representative of pregnant women in Hungary, particularly those with lower socioeconomic resources or lower health literacy. In addition, 21 questionnaires with insufficient completion were excluded from the analysis. Although the reasons for non-completion were not available, differential questionnaire completion cannot be ruled out and may have introduced additional selection bias.

Although the subscores for the four knowledge domains were standardised and combined into an equally weighted composite score, the psychometric properties of the instrument have not been comprehensively validated. Response and social-desirability bias cannot be entirely excluded for self-reported variables. In addition to variables included in the regression models, other potentially relevant factors—such as previous healthcare experiences, use of health information sources, and prior pregnancy-related knowledge—may have influenced reproductive health knowledge and preconception preparedness but could not be fully controlled for. Furthermore, the number of domain-specific and subgroup comparisons may have increased the risk of type I errors; therefore, these findings should be considered exploratory and interpreted cautiously.

A further limitation concerns the operationalisation of the primary outcome. The composite measure combined three knowledge-oriented domains with a preconception preparedness domain that included self-reported behavioural and healthcare-use indicators, such as lifestyle modification, preconception consultation, screening, and folic acid or prenatal vitamin use. Consequently, the overall score should not be interpreted as a pure or unidimensional measure of reproductive health knowledge. The findings should therefore be interpreted as associations with a study-specific composite indicator of reproductive health knowledge and preconception preparedness. Although standardisation to a 0–100 scale and equal weighting of the four domains provided a transparent and predefined scoring approach, this weighting was conceptual rather than psychometrically derived. This may limit the construct validity and interpretability of the composite score. The questionnaire was developed specifically for the present study based on reproductive health-related protective and risk factors identified in the literature; however, it was not subjected to formal psychometric validation. Consequently, the reported internal consistency coefficients should be interpreted only as indicators of internal consistency and not as evidence of content, construct or criterion validity. Future research should use validated multidimensional instruments or separately analyse knowledge and preconception practice outcomes.

Despite these limitations, the findings contribute to a better understanding of the associations between health literacy and reproductive health knowledge and preconception preparedness among pregnant women. They provide a basis for future larger, longitudinal studies using validated multidimensional measurement instruments and more diverse, population-based samples to further examine these associations and their temporal relationships.

## 5. Conclusions

The present study identified an association between objective functional health literacy and the composite measure of reproductive health knowledge and preconception preparedness among pregnant women in Hungary. In the fully adjusted hierarchical regression model, higher NVS scores were associated with higher scores on this composite outcome, whereas subjective health literacy measured by BRIEF was not statistically significantly associated with the outcome after simultaneous adjustment for both health literacy measures and relevant sociodemographic and obstetric characteristics. The addition of the two health literacy measures increased the explained variance by 7.6 percentage points, with the final model explaining 15.8% of the variance. These findings suggest that objective and subjective health literacy capture partly different aspects of health-related competencies and should not be considered interchangeable measures.

The findings may inform future research on health literacy-sensitive antenatal education, particularly approaches addressing specific reproductive health knowledge gaps and preconception preparedness. Health literacy assessment and attention to domain-specific knowledge may provide potentially useful directions for further investigation of tailored antenatal education. However, the present study did not evaluate the effectiveness of screening strategies, educational interventions, behaviour change, or maternal and neonatal outcomes. Therefore, the findings should not be interpreted as evidence that health literacy assessment or tailored education improves reproductive health knowledge and preconception preparedness or health outcomes.

The cross-sectional design precludes conclusions regarding temporal ordering or causality, and the primary outcome was based on a study-specific, researcher-developed composite measure that has not undergone formal psychometric validation. Larger longitudinal and intervention studies using validated multidimensional instruments and more diverse samples are needed to further examine the temporal relationships among health literacy, reproductive health knowledge, and preconception preparedness and to determine whether health literacy-sensitive educational approaches can improve these outcomes.

## Figures and Tables

**Table 1:** Socio-demographic indicators of the sample (*N* = 346).

Variable	Category	Value
Gestational age (weeks, M ± SD; min-max)		28.38 ± 7.65; 13–40
Age (years, M ± SD; min-max)		30.49 ± 4.33; 20–41
Educational attainment (*n* (%))	University/college	249 (71.97)
	Secondary school/grammar school	91 (26.30)
	Vocational secondary school/vocational training school	6 (1.73)
	Primary education (8 years) or less	0 (0.00)
Residence (*n* (%))	Capital city/county seat	131 (37.86)
	Town	118 (34.10)
	Village/rural municipality	97 (28.03)
Self-rated financial situation (*n* (%))	Able to afford all necessary expenses and save money	169 (48.84)
	No financial difficulties, but unable to save money	145 (41.91)
	Just able to cover expenses	25 (7.23)
	Unable to cover expenses	7 (2.02)
Number of children (already born) (*n* (%))	0	206 (59.54)
	1	88 (25.43)
	2	32 (9.25)
	3	10 (2.89)
	≥4	10 (2.89)

M = mean; SD = standard deviation; n = number of respondents.

**Table 2:** Results of analyses examining associations between reproductive health knowledge-preconception preparedness and health literacy.

Reproductive Health Knowledge and Preconception Preparedness Domain	Subjective Health Literacy (BRIEF)	Objective Functional Health Literacy (NVS)
Nutrition knowledge	H(2) = 4.31; *p* = 0.116	H(2) = 38.82; *p* < 0,001
Smoking-related knowledge	H(2) = 2.81; *p* = 0.245	H(2) = 6.69; *p* = 0.030
Conscious preparation for pregnancy	H(2) = 2.12; *p* = 0.340	H(2) = 1.52; *p* = 0.470
Knowledge of risks associated with maternal and paternal age	H(2) = 16.17; *p* < 0.001	H(2) = 5.48; *p* = 0.062
Overall reproductive health knowledge	F(2, 343) = 4.93; *p* = 0.008	F(2, 343) = 9.618, *p* < 0.001

**Table 3:** Hierarchical multiple linear regression model of factors associated with reproductive health knowledge and preconception preparedness.

Predictor	Step 1: Control Variables				Step 2: Full Model				
	B	SE	β	*p*	B	SE	β	*p*	95% CI for B
Age	0.111	0.171	0.052	0.51	0.145	0.172	0.079	0.345	−0.196 to 0.485
Higher education	1.509	1.716	0.069	0.330	0.866	1.736	0.031	0.563	−2.267 to 4.300
Town	0.606	1.818	0.028	0.759	0.177	1.735	0.006	0.946	−3.232 to 3.556
Village/rural municipality	−3.586	1.854	−0.165	0.066	−4.046	1.804	−0.176	0.029	−7.973 to −0.435
Gestational age	−0.007	0.106	−0.006	0.961	−0.013	0.104	−0.013	0.842	−0.238 to 0.182
Parity	−1.587	0.787	−0.147	0.060	−1.530	0.863	−0.142	0.056	−3.237 to 0.042
Planned pregnancy	−0.636	2.667	−0.033	0.881	−0.838	2.586	−0.021	0.697	−5.525 to 4.950
Good financial situation	−1.273	2.826	−0.048	0.615	−0.776	2.744	−0.029	0.760	−6.354 to 4.406
Subjective health literacy (BRIEF)	—	—	—	—	0.244	0.203	0.088	0.227	−0.177 to 0.654
Objective functional health literacy (NVS)	—	—	—	—	1.732	0.553	0.271	0.001	0.667 to 2.627
R^2^	0.078				0.151				
Adjusted R^2^	0.036				0.103				
ΔR^2^	—				0.077				
F	1.664			0.106	—				
ΔF	—				7.121			0.003	
Durbin–Watson	—				2.096				

Note. The dependent variable was the overall reproductive health knowledge and preconception preparedness score. In Step 1, age, educational attainment, place of residence, gestational age, parity, pregnancy planning status, and financial situation were entered as control variables. In Step 2, subjective health literacy (BRIEF score) and objective functional health literacy (NVS score) were entered simultaneously as continuous variables. Reference categories were secondary education for educational attainment, capital city/county seat for place of residence, unplanned/other for pregnancy planning status, and average/poor for financial situation. B = unstandardised regression coefficient; SE = standard error; β = standardised regression coefficient; CI = confidence interval; R^2^ = coefficient of determination; ΔR^2^ = change in explained variance; ΔF = change in the F statistic associated with the addition of Step 2 predictors.

## Data Availability

The data presented in this study are available on request from the corresponding author. The datasets generated and analysed during the current study are not publicly available due to privacy and ethical restrictions, as they contain sensitive information collected from women, including pregnant participants. Data may be made available from the corresponding author upon reasonable request and subject to institutional and ethical approval.

## Supplementary Materials

The following supporting information can be downloaded at https://www.mdpi.com/article/10.3390/medsci14060692/s1, Table S1: Sensitivity analyses using alternative specifications of the composite outcome.

## Abbreviations

The following abbreviations are used in this manuscript:

BRIEF	Brief Health Literacy Screening Tool
COM-B	Capability, Opportunity, and Motivation, which interact to generate Behavior B
NVS	Newest Vital Sign
RHK	Reproductive health knowledge
VIF	Variance inflation factor
