# Supplementary material for: Associations of Objective Functional and Subjective Health Literacy with Reproductive Health Knowledge and Preconception Preparedness Among Pregnant Women in Hungary
Source: Med Sci (Basel). 2026 Oct 21;14(6):692. doi: 10.3390/medsci14060692 (PMC13707322; doi:10.3390/medsci14060692)
Supplement: Supplementary file 1 [file medsci-14-00692-s001.zip › medsci-4573569-supplementary.pdf]

Supplementary material

Supplementary Table S1. Sensitivity analyses using alternative specifications of the composite outcome

	**Main model**	**Sensitivity 1**	**Sensitivity 2**
N	346	346	346
Step 1 R^2	.078	.095	.181
Step 2 R^2	.151	.202	.207
ΔR^2	.077	.106	.048
NVS B	1.732	2.274	1.238
NVS β	.271	.377	.161
NVS p	.001	.001	.001
BRIEF B	.244	.288	.362
BRIEF β	.088	.091	.108
BRIEF p	.227	.079	.273
